# Effect of the Pulsed Electric Field Treatment on Physical, Chemical and Structural Changes of Vacuum Impregnated Apple Tissue in Aloe Vera Juices

**DOI:** 10.3390/foods12213957

**Published:** 2023-10-29

**Authors:** Magdalena Trusinska, Federico Drudi, Katarzyna Rybak, Urszula Tylewicz, Malgorzata Nowacka

**Affiliations:** 1 Department of Food Engineering and Process Management, Institute of Food Sciences, Warsaw University of Life Sciences—SGGW, Nowoursynowska 159c, 02-776 Warsaw, Poland; magdalena_trusinska@sggw.edu.pl (M.T.); katarzyna_rybak@sggw.edu.pl (K.R.); 2 Department of Agricultural and Food Sciences, University of Bologna, Piazza Goidanich 60, 47521 Cesena, Italy; federico.drudi3@unibo.it (F.D.); urszula.tylewicz@unibo.it (U.T.); 3 Interdepartmental Centre for Agri-Food Industrial Research, University of Bologna, Via Quinto Bucci 336, 47521 Cesena, Italy

**Keywords:** non-thermal processing, impregnation effectiveness, physical properties, structure, chemical changes, bioactive compounds, cell viability, plant tissue

## Abstract

Vacuum impregnation (VI) stands as a diffusion-driven food processing method that has found recent application within the food industry, particularly for the cold formulation of fortified food products. Pulsed electric field (PEF) treatment can affect the food structure, influencing therefore the mass transfer phenomena during the further processing. Thus, the study aimed at investigating the effect of PEF treatment on selected physicochemical properties of vacuum-impregnated apples. Apple slices were vacuum impregnated with aloe vera juice solution with or PEF treatment at different intensities (125, 212.5 or 300 V/cm). The PEF was applied as a pretreatment—applied before the VI process as well as posttreatment—applied after the VI process. The VI process with aloe vera juice resulted in a sample weight increase of over 24% as well as structural changes, partial cell viability loss and color alteration. In addition, the decrease of bioactive compounds was observed, while antioxidant activity remained at a similar level as in raw material. PEF treatment adversely affected vacuum impregnation efficiency, causing microstructural changes and cell viability loss. Additionally, chemical composition modifications were evident through thermogravimetric analysis (TGA) and Fourier Infrared Spectroscopy (FTIR) analyses. Tissue hardness decreased significantly due to structural damage and caused high leakage from plant tissue, which resulted in hindering saturation with aloe vera juice during the VI process. Additionally, reduced bioactive substance content after PEF treatment was observed and the VI process did not restore apple samples of the bioactive compounds from aloe vera juice.

## 1. Introduction

Vacuum impregnation (VI) is a diffusion-based food processing technique that has recently been used in the food industry for the development of functional foods [1]. VI works by a mechanically induced pressure difference between porous foods that are submerged in a solution surrounding them. First, the pressure in the food-liquid system is reduced (vacuum step), allowing the native gases in the pores to expand and move away, while the product pores are also expanded. When mechanical equilibrium is reached, atmospheric pressure is restored (relaxation step). Doing so, the tissue relaxes and the remaining gas contracts, while the external solution fills the pores, thanks to the action of the hydrodynamic mechanism and the deformation-relaxation phenomena [2,3].

There are many parameters that affect impregnation. Vacuum pressure correlates negatively with impregnation yield, deleterious effects on the overall structure of the product can increase when extremely low pressure is achieved. Longer holding and relaxation times generally increase impregnation yield, while solution viscosity can be challenging and hardly be introduced in the food matrix [4]. In any case, all process parameters must be investigated for each specific product, since the type, dimension and number of pores can vary greatly among different plant food matrices [5].

The composition of the solution, e.g., bioactive compounds content and solution concentration plays a crucial role in the development of functional and fortified food products. The most studied compounds to be added in the impregnating solutions have been antioxidants [6], minerals such as calcium [7] or iron [8], vitamins such as ascorbic acid [9] or tocopherol [10] and probiotics [11].

Recently the application of juices, both concentrated or diluted, has also been studied. In particular, apples have been enriched with the phenolic compounds from the blueberry juice [12] and grape juice concentrate [13]. In addition, apple juice [14] and mandarin juice [15] were used as a medium for probiotic introduction into the apple tissue. Moreover, aloe vera gel has been used to impregnate apple slices by De Rossi et al. [16].

Aloe vera is a perennial plant with fleshy leaves and a cactus-like form that belongs to the Asphodelaceae family or a broadly circumscribed family called Liliaceae [17]. It is very well known for its curative and therapeutic properties such as lowering cholesterol and triglyceride levels in human blood, anti-inflammatory and anti-biotic properties, beneficial effects against gastrointestinal, renal and cardiovascular diseases [18,19,20]. Aloe vera offers numerous potential health benefits. It has been seen to enhance cardiac contraction strength, lower cholesterol levels, reduce triglyceride levels, reverse existing atheromatous cardiovascular disease, stimulate the regeneration of cells responsible for insulin synthesis and release and provide long-lasting blood glucose control properties. It is used for ulcers, gastrointestinal problems and kidney ailments [21]. Nowadays, the market for products derived from this source is worth hundreds of millions of dollars worldwide and includes various products such as gels, juices, dried supplements and more [22].

Pulsed electric field (PEF) is a technology based on the application of short pulses (generally µs) of high voltage to conductive products that can affect the cell membrane through the electroporation effect, that is, the creation of pores of reversible or permanent nature [23]. It is considered a non-thermal treatment often used to improve mass transfer in vegetable products [24]. Recently, it has also been studied as a pretreatment along with vacuum impregnation of potatoes [25], strawberries [26] and seafood [27]. Thus, the aim of this work was to determine the effect of pulsed electric field (PEF) treatment on selected physicochemical and structural properties of apples subjected to vacuum impregnation in Aloe vera juice. PEF pretreatment with different electric field strengths (125, 212.5 or 300 V/cm) was performed before or after the VI process to verify the effects on fruit tissue properties.

## 2. Materials and Methods

### 2.1. Materials

The research material consisted of certified organic apples of the Golden Delicious variety (12 ± 0.5° Brix), purchased in one batch from a local store in Cesena (Italy). Before the tests, fruits were stored at a temperature of 4 ± 1 °C and a relative humidity of 80%, for not longer than 10 days. The apples were washed in tap water and cut into 5 mm thick slices, and then the seeds and peel were removed with the cork borer and sharp knife, respectively.

Aloe vera juice, used to perform vacuum impregnation, was obtained from Aloe Barbadensis Miller leaves from controlled organic farming—Alba Aloe Vera Organic 99.9% Bio by Benessence (1° Brix). In order to avoid the phenomenon of osmosis during vacuum impregnation, an isotonic solution was prepared by dissolving an appropriate amount of trehalose (EXACTA + OPTECH Labcenter S.p.A., San Prospero, Italy) in aloe vera juice (to obtain isotonic solution around 11.5 g of trehalose dissolved in 88.5 g of aloe vera juice).

### 2.2. Technological Processing

#### 2.2.1. Pulsed Electric Field Treatment

An S-P7500 pulse generator (Alintel SRL., Bologna, Italy) with a maximum output voltage and current of 8 kV and 60 A, respectively, was used to perform the pulsed electric field treatment. Six apple slices were placed in a chamber equipped with two parallel electrodes. The distance between the electrodes was set to 12 cm. The chamber was filled with tap water at a temperature of 25 ± 1 °C and initial electrical conductivity, measured with an EC-meter Mod. Basic 30 (Crison, Barcelona, Spain), of 471  ±  5 μs/cm. Based on preliminary tests (to obtain reversible and irreversible electroporation), the following process parameters were selected: electrode voltage 1.5 kV, number of pulses 60, frequency 100 Hz, pulse width 0.1 ms and electric field strength 125, 212.5 or 300 V/cm. Table 1 presents the electrical capacitance and resistance of the samples subjected to the PEF treatment, on the basis that the process might be described as reversible or irreversible.

#### 2.2.2. Vacuum Impregnation Process (VI)

**Figure 1:**
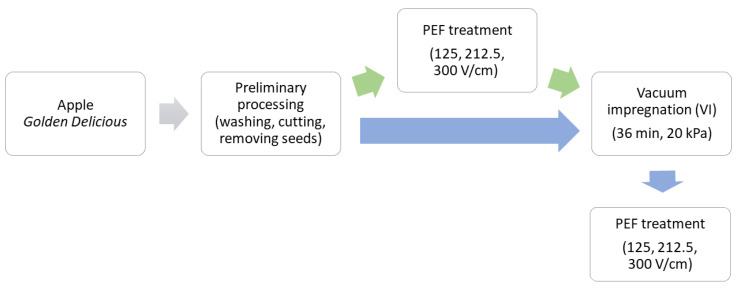
Scheme of the vacuum-impregnation processing of apples with the use of PEF treatment of different intensities (125, 212.5 and 300 V/cm) applied before or after vacuum impregnation.

The VI process was applied before or after the PEF treatment (Figure 1) and 6 different samples after the processing were obtained (Table 2).

The vacuum impregnation process was carried out in a closed chamber connected to a vacuum pump and an automatic vacuum control system (AVCS, S.I.A., Bologna, Italy) all maintained at a consistent temperature of 25 ± 1 °C. Weighed apple slices were placed into 800 cm^3^ beakers on a metal frame, preventing contact of the samples to each other. Subsequently, the solution has been added into beakers, with a solution-to-mass sample ratio of 7:1 (V/m). The VI treatment lasted 36 min, with a gradual increase of the vacuum lasting 8 min (100–80 kPa for 2 min, 80–60 kPa for 2 min, 60–40 kPa for 2 min, 40–20 kPa for 2 min), keeping the sample under pressure of 20kPa (absolute pressure)—10 min, and gradual restoration of atmospheric pressure—8 min. The relaxation time after vacuum impregnation was 10 min. Afterward, the apple slices were withdrawn from the solution, gently dried on filter paper and their mass was recorded. Six replicates were performed for each sample. The weight gain (WG) during the process was computed using the formula described by Tylewicz et al. [28]:

WG = 100·(*m* − *m*_0_)/*m*_0_, (1)

where

*m*—mass of the sample after vacuum impregnation,

*m*_0_—mass of the sample before vacuum impregnation.

### 2.3. Analytical Methods

#### 2.3.1. Water Content and Water Activity

The water content was determined using gravimetric method [29]. The crushed samples were dried in a laboratory dryer at a temperature of 70 °C for 48 h and then water content was calculated on the basis of dry matter content. The determination was performed three times for each sample.

The water activity of the samples was measured with an AquaLab Series3TE device (Decagon Devices, Inc., Pullman, Washington, DC, USA) with an accuracy of ±0.001 [30]. The measurement was performed in duplicate for each sample at a temperature of 25 ± 1 °C.

#### 2.3.2. Color Measurements

The color was measured using a Colorflex colorimeter (Hunterlab, Reston, VA, USA) in the CIE L*a*b* system, where: L*—brightness, parameter a*—value of the color chromaticity coordinate from green to red and parameter b*—value of the color chromaticity coordinate from blue to yellow. Before measurement, the device was calibrated using black and white standards. The measurement was made ten times for each material using a D65 medium daylight source and a standard 10° observer. For each sample, the total color differences (ΔE) and the browning index (BI) were calculated in relation to fresh apple tissue [31] according to following equations:

(2) $$∆E=\sqrt{{\left(∆L^{*}\right)}^{2}+{\left(∆a^{*}\right)}^{2}+{\left(∆b^{*}\right)}^{2}},$$

where: ∆L*—difference in brightness values between a processed apple and a fresh one, ∆a*—difference in the value of the a* parameter between a processed apple and a fresh one, ∆b*—difference in the value of the b* parameter between a processed apple and a fresh one;

(3) $$BI=100\cdot \frac{x-0.31}{0.172},$$

where: $x=\frac{a^{*}+1.75\cdot L^{*}}{5.645\cdot L^{*}+a^{*}-3.012\cdot b^{*}}$.

#### 2.3.3. Texture Measurements

The texture of apples was analyzed using a Texture Analyser TA HDi500 (Stable Micro Systems, Surrey, UK). Textural parameters were evaluated through a compression test. Apples placed on the platform were compressed at random points using an aluminum cylinder probe with a diameter of 5 mm moving at a head speed of 1 mm/s. The process was stopped after obtaining 50% of the sample height [32]. The maximum force needed to cause a deformation of the material was read from the obtained graphs. Twenty replicates were performed for each sample.

#### 2.3.4. Structure Analysis

A scanning electron microscope Phenom XL (Phenom World, Eindhoven, The Netherlands) was used to observe changes in the internal structure of the apple. The material was subjected to a freeze-drying process to remove water from the tissues. The slices were placed in the freezer at −40 °C for 48 h. Freeze drying was carried out in a Lio2000 freeze dryer (Cinque Pascal S.r.l., Milan, Italy), with drying parameters: a shelf temperature of 25 °C, a pressure of 22 Pa, a condenser temperature of −47 °C and a time of 48 h. After the process, the material was placed in light and air barrier packaging. A small piece of freeze-dried plant tissue, cut with a razor blade, was attached to carbon tape and to a metal table. The samples were sputter coated (Cressington 108 auto, Cressington Scientific Instruments, Watford, UK) 5 nm gold layer for 25 s to increase their electrical conductivity. Observations under the microscope were carried out at a voltage of 10 kV and a vacuum of 60 Pa. Photos were recorded at 200× magnification.

#### 2.3.5. Assessment of Cell Viability

The assessment of cell viability was performed to evaluate cell membrane integrity after PEF and VI processes. This parameter is important to evaluate the damage to the cells as well as electrolyte leakage after processing, which helps in explaining the changes that occur in the plant tissue. This, fluorescein diacetate (FDA, Sigma-Aldrich, Milano, Italy, λex 495 nm, λem 518 nm) was used to determine cell viability (green-living cell) [33]. A stock solution of 10^−4^ FDA was prepared in water and stored at 4 °C. The apple pieces 1 mm thick were incubated in the dark at 25° C for 5 min in a dye solution prepared with an isotonic concentration of sucrose. The incubated samples were rinsed with deionized water and examined under fluorescent light in a Nikon upright microscope (Eclipse TieU, Nikon Co, Tokyo, Japan), equipped with a Nikon digital video camera (digital sight DS-Qi1Mc, Nikon Co, Tokyo, Japan), at a magnification of 10. Around 6 to 8 photos were taken for each sample.

#### 2.3.6. Assessment of Bioactive Compounds

##### Extract Preparation

For the chemical determinations the samples were subjected to the freeze-drying process (parameters of the process are described in Section 2.3.4), to standardize samples and improve extractability. The dried apples were ground in an A 11 basic analytical grinder (Ika-Werke, Staufen, Germany). Approximately, 0.3 g of the tested material was weighed into the falcon and 10 mL of extraction reagent (80% ethyl alcohol and 0.1 M hydrochloric acid, 85:15 vol/vol) was added. Extraction was carried out for 12 h at room temperature on a Multi Reax shaker (Heidolph Instruments, Schwabach, Germany) protected from light. The solution was centrifuged in a MegaStar 600 laboratory centrifuge (VWR, Leuven, Belgium, 4350 rpm/min, 2 min). The extraction procedure was performed twice for each sample.

##### Determination of Total Polyphenol Content (TPC)

The content of polyphenols in apples was determined by the spectrophotometric method using a color reaction with the Folin–Ciocalteau reagent according to the methodology described by Bochniak-Niedźwieck et al. [34]. The extracts were diluted twice with distilled water. Reactions were performed in 96-well plates. A total of 40 µL of Folin–Ciocalteau reagent diluted 5 times was added to 10 µL of the extract, and 250 µL of 7% sodium carbonate solution was added after 3 min. The solution was incubated for 60 min at room temperature without access to light. Absorbance measurement at 750 nm was performed using a Multiskan Sky plate reader (Thermo Electron Co., Waltham, MA, USA). The blank was prepared in the same way, with the extract replaced by the extraction reagent. Two repetitions were performed for each of the tested extracts. In order to quantitatively determine the content of polyphenols, a calibration curve was prepared for chlorogenic acid (Sigma Aldrich, Switzerland) in the range of 0–100 g/mL and the results were expressed as the amount of mg of chlorogenic acid in 100 g of dry substance.

##### Determination of Vitamin C

The vitamin C content was determined using the UPLC-PDA system (WATERS Acquity H-Class, Milford, MA, USA). The measurement was made according to the method presented by Beya et al. [35]. A total of 10 mL of cold extraction solution (3% metaphosphoric acid, 8% acetic acid and 1 mM EDTA solution) was added to 0.05 g of material and stirred for 10 min on a laboratory shaker. The solution was centrifuged (6000 rpm, 4 °C, 5 min) and the supernatant was filtered through syringe filters (0.22 μm PTFE) and diluted twice with eluent (Milli-Q water with 0.1% formic acid). A WATERS Acquity UPLC HSS T3 chromatographic column (2.1 × 100 mm, 1.8 μm; Waters, Ireland) was used for the determination. The flow of the mobile phase was 0.25 mL/min and the temperature of the column thermostat and samples were 25 and 4 °C, respectively. The spectrum was analyzed at a wavelength of 245 nm. The content of L-ascorbic acid was calculated based on the calibration curve of the L (-) ascorbic acid analytical standard. The analysis was performed twice for each sample.

##### Determination of Antioxidant Activity (AA)

The measurement of the antioxidant activity of the analyzed samples was based on determining the ability of the sample solution to reduce the ABTS^•+^ cation radical. The starting solution was prepared by dissolving 38.4 mg of 2,2′-Azino-bis(3-ethylbenzthiazoline-6-sulfonic acid) and 6.6 mg of potassium persulfate) in 10 mL of distilled water. The solution was mixed and left at 4 °C for 12 h. The working solution was prepared immediately before the analysis by diluting the starting solution 100 times with an 80% ethanol solution. 10, 20, 30 and 40 µL of the sample extract and 3 mL of the radical solution were added to the reaction tubes. After 6 min, the absorbance was measured at a wavelength of 734 nm (Heλios Thermo Electron 7.03 spectrophotometer, Thermo Electron Corporation, Waltham, MA, USA) [36]. Antioxidant activity was determined on the basis of the decrease in the absorbance of the radical solution in the presence of an antioxidant and expressed in mg of Trolox/g of dry sample substance. The assay was performed in duplicate.

#### 2.3.7. Fourier Infrared Spectroscopy (FTIR)

Measurements of infrared spectra were performed with a Cary 630 spectrometer (Agilent Technologies Inc., Santa Clara, CA, USA), with a single reflection diamond interface (ATR—attenuated total reflectance). The spectra were recorded in the wavenumber range 4000–650 cm^−1^, with the number of scans 32, at a resolution of 4 cm^−1^ [37]. Data were assessed using MicroLab FTIR software (version 5.7). Three measurements were performed for each sample.

#### 2.3.8. Thermogravimetric Analysis (TGA)

The thermal stability and decomposition of the samples were determined using a TGA/DSC 3+ thermogravimeter (Mettler Toledo, Greifensee, Switzerland). A total of 5 to 7 mg of the dried material was weighted in 70 µL Al_2_O_3_ crucibles and heated from 30 to 600 °C at a rate of 5 °C/min in a nitrogen atmosphere (50 mL/min) [38]. The TG and DTG curves were obtained from the differential values of the dependence of mass loss on temperature. Thermograms TGA/DTG were evaluated using the STAR software (version 16.10) from Mettler Evaluation. Two replicates were performed for each sample.

### 2.4. Statistical Methods

STATISTICA 13 software (StatSoft Inc., Tulsa, OK, USA) was used for statistical analysis. The relationships between the tested properties of apples and the applied processing were analyzed using a one-way ANOVA analysis of variance. In order to identify which samples were statistically significantly different from each other, multiple comparisons analysis using Tukey’s test was used. Statistical inference was conducted at the significance level α = 0.05. In order to comprehensively assess the obtained results, based on the analyzed physicochemical properties, a cluster analysis and principal components analysis were performed. Ward’s method and the Euclidean distance were used. The results of the cluster analysis were presented on the dendrogram.

## 3. Results and Discussion

### 3.1. Vacuum Impregnation Process Combined with PEF Treatment of Apples

**Figure 2:**
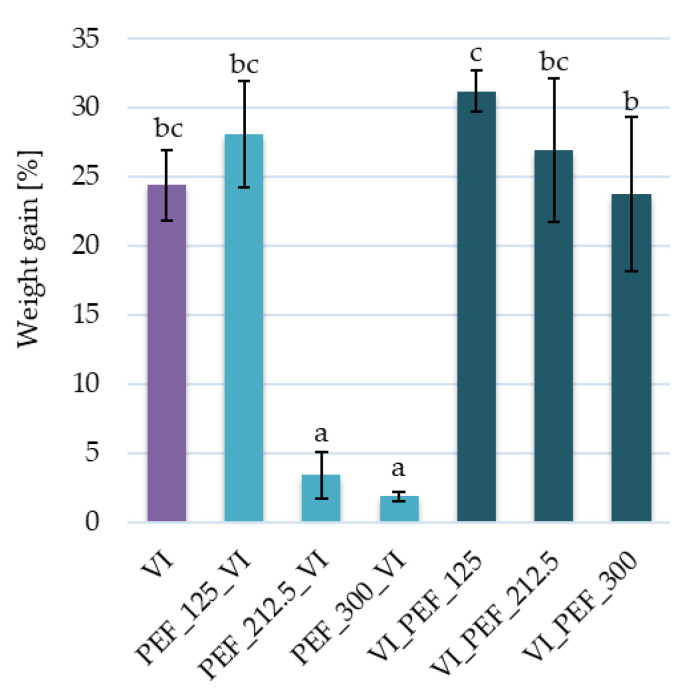
Weight gain of vacuum-impregnated apples obtained with or without the PEF treatment of different intensities (125, 212.5, 300 V/cm) applied before or after vacuum impregnation. The same letters above the columns indicate homogeneous groups that do not differ statistically from each other (α = 0.05).

The impregnation yield expressed as weight gain is dependent on many factors, among which the structure of the material, mechanical properties and process parameters are considered as the most essential. Porosity is one of the most important variables of a vacuum-impregnated material affecting the process efficiency, as it indicates the void space for the inflow of the impregnating solution [39]. Apple is characterized by a relatively high porosity (about 18–27%) which makes it a fruit that is often used in vacuum impregnation studies [4,40,41,42]. In the present study, VI of apples in aloe vera juice solution resulted in a weight gain of 24.4 ± 2.5% (Figure 2), whereas in the literature the following results can be found: 15.17 ± 0.82 [43], 23.4 ± 0.5% [44], 27% (vacuum pressure of 300 mbar) or 31% (vacuum pressure of 100 mbar) [45]. When PEF at low electric field strength (125 V/cm) was used the mass gain was on a similar level as the VI sample, which was probably related to small changes caused by PEF treatment and reversible electroporation. However, when PEF at 212.5 or 300 V/cm was applied before VI, the weight gain amounted to 3.42 ± 1.68 and 1.87 ± 0.32%, respectively, and was significantly lower than in the other cases. It may have been caused by the irreversible electroporation during PEF treatment, which could lead to significant leakage of cellular juice from the apples to the solution. However, the opposite result was noted by Mashkour et al. [46], who analyzed the effect of vacuum impregnation of potatoes with PEF pre-treatment on iron enrichment. The study showed that weight gain increased with the increasing PEF intensity and number of pulses. It was explained by the electroporation mechanism, which resulted in easier removal of air from the tissue with higher permeability of cell membranes during the reduced pressure stage and easier penetration of the impregnating solution during the relaxation stage [46].

**Figure 3:**
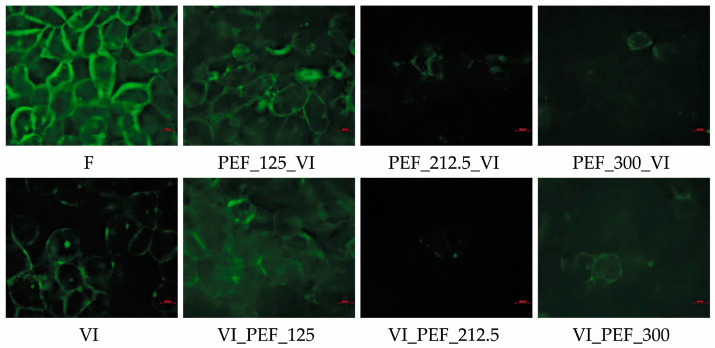
Cell viability of parenchyma apple tissue stained with FDA subjected to vacuum impregnation with or without the use of PEF treatment at different intensities (125, 212.5, 300 V/cm) applied before or after vacuum impregnation compared to the fresh one.

Figure 3 shows microphotographs of apple tissue stained with FDA solution aiming at assessing cell viability. FDA is able to pass into cells through intact membranes, where it is hydrolyzed by esterases to a fluorescent compound that cannot pass through the membrane, considered to be functional only in the viable cells [47]. The VI resulted in the preservation of cell viability, as it can be observed by the green fluorescence of the living cells. The cell viability retention after the vacuum impregnation was observed also in other studies [44,48,49,50]. However, several parameters can affect cell viability such as the pH of impregnating solution or the concentration of substances dissolved in the solution. For example, Mauro et al. [51] showed that vacuum impregnation in solutions with sucrose and calcium lactate concentrations of 20 and 2%, respectively, resulted in the preservation of the cellular structure, but increased concentrations of these compounds resulted in cell death. In turn, Tappi et al. [44] observed that impregnating apple tissue with sucrose alone or sucrose with calcium lactate solutions resulted in the preservation of cell viability, while the addition of ascorbic acid to the solution resulted in cell death. The researchers suggested that ascorbic acid may have remained in the intercellular space and was exposed to oxygen, which caused its oxidation leading to loss of cell viability.

Apple samples subjected to PEF at 125 V/cm and vacuum impregnation showed partial preservation of cell viability, while PEF at higher intensities (212.5 or 300 V/cm) combined with vacuum impregnation led to almost complete loss of cell viability (Figure 3). This phenomenon was observed independently of the PEF position, before or after the VI process. These results are in line with those obtained by Tylewicz et al. [52], who investigated the effect of PEF pre-treatment at 100 or 200 V/cm on the properties of fresh and osmotically dehydrated kiwi fruit. The researchers observed that PEF pre-treatment at 100 V/cm led to the partial preservation of cell viability of osmotically dehydrated fruit, while higher PEF intensity (200 V/cm) caused cell death [52]. Similar behavior was observed in the preservation of cell viability in the PEF pre-treated (100 or 200–400 V/cm) osmodehydrated strawberries [53]. Whereas, Dellarosa et al. [51] found that the application of PEF at 250 or 400 V/cm resulted in a significant loss of cell viability of apple tissue (irreversible electroporation), while when PEF was applied at 100 V/cm, the result was comparable to fresh apple, indicating that only reversible electroporation occurred, which did not result in loss of cell viability. From the images obtained in the present study (Figure 3), it can be concluded that PEF at 212.5 or 300 V/cm induced irreversible electroporation, which resulted in permanent perforation of the cell membrane and led to cell death, whereas PEF at 125 V/cm induced reversible electroporation. Despite the limitation of the method used to assess cell viability, which is the analysis of the small area of material, the results obtained in other analyses (weight gain, hardness and color change) seem to confirm these observations. A similar effect was noticed in kiwi fruit subjected to osmotic dehydration, where a greater loss of tissue hardness was noticed when PEF treatment led to irreversible electroporation [52].

### 3.2. The Effect of Vacuum Impregnation of Apples in Aloe Vera Juice on the Change of Physical Properties

#### 3.2.1. Changes in Water Content and Water Activity in Apples

**Figure 4:**
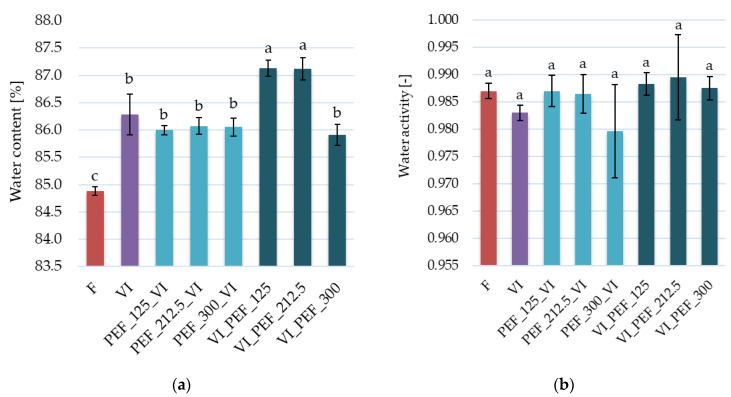
Water content (**a**) and water activity (**b**) of fresh and vacuum-impregnated apples obtained with or without the use of PEF treatment of different intensities (125, 212.5, 300 V/cm) applied before or after vacuum impregnation. The same letters above the columns indicate homogeneous groups that do not differ statistically from each other (α = 0.05).

In Figure 4 water content and water activity of the samples are presented. The water content of fresh apples was 84.88 ± 0.08 g/100 g of product, while as a consequence of vacuum impregnation, it significantly increased to 86.29 ± 0.37 g/100 g of product. Similarly, de Lima et al. [54] noted that the moisture content of vacuum-impregnated pineapples was higher than fresh samples which was explained by water uptake during the treatment. On the other hand, a different result was reported by Nawirska-Olszańska et al. [55], who did not observe a statistically significant change in water content for fresh and vacuum-impregnated black chokeberry in freshly pressed apple and pear juice (12.4° Brix). The differences in obtained results may be due to the use of different raw materials, impregnating solutions and process conditions.

Vacuum impregnation combined with PEF treatment, regardless of PEF intensity and the treatment order, also resulted in a statistically significant increase in water content in relation to the fresh material. Additionally, for PEF treatment applied after vacuum impregnation, water content increased significantly to 87.13 ± 0.15 g/100 g and 87.12 ± 0.20 g/100 g of product when the PEF intensity of 125 or 212.5 V/cm was applied, respectively.

Water activity of food products is determined in order to evaluate their susceptibility to microbial growth. A food material is considered microbiologically stable if the value of water activity is below 0.6, whereas the value of water activity exceeding 0.95 enables the growth of the majority of microorganisms [55]. From Figure 4b, it might be concluded that neither vacuum impregnation alone nor the combination of vacuum impregnation and PEF did not cause a significant change in water activity in comparison to the fresh apple. This result is in line with the research of Hinestroza-Córdoba et al. [56], in which no significant difference between fresh and vacuum-impregnated samples of lulo fruit was obtained. Because of high values of water activity (between 0.98 and 0.99) both fresh and vacuum-impregnated fruits are highly perishable and potentially hazardous products [7] that need to be further processed to obtain microbiologically stable products.

#### 3.2.2. Changes in Apple Color

Color is a crucial food property that has a key role in the assessment of food quality and if not optimal can negatively affect consumers’ visual perception of a food product [57]. The results of L*, a* and b* parameters, as well as calculated values of ΔE and BI are presented in Table 3.

The value of the L* parameter for the fresh fruit amounted to 78.63 ± 2.22 and was significantly higher than for the other samples, which ranged from 44.43 ± 5.43 to 53.09 ± 2. The lower value of the L* parameter of vacuum-impregnated apples may be due to the introduction of the aloe vera juice solution into the apple pores, but also to the different way in which light is reflected from the surface of the fresh fruit with a porous structure from the vacuum-impregnated fruit, whose pores are filled with liquid. In fact, the determination involves measuring the radiation reflected from the surface of the material. The solution filling the pores of the vacuum-impregnated tissue can absorb part of the radiation so that a smaller part of it is reflected from the surface, which is recorded by the measurement device [58]. A similar observation was conducted for apples impregnated in a sucrose solution with ascorbic acid, green tea extract or a combination of these substances [6], or impregnated apples in a sucrose solution with ascorbic acid, calcium lactate or their combination [44]. As a result of the combination of PEF treatment and vacuum impregnation, the brightness did not change significantly compared to material subjected to vacuum impregnation alone. Furthermore, it was observed that samples treated with PEF at 125 V/cm before or after vacuum impregnation had significantly lower L* values than samples treated with PEF at 212.5 or 300 before vacuum impregnation.

The parameter a*, which relates to color changes ranging from green to red, for the untreated apple was 0.92 ± 0.23, for the apple after VI alone 0.77 ± 1.10 and when PEF treatment was applied, the parameter a* had a value ranging from 0.10 ± 1.01 (PEF after VI, 300 V/cm) to 7.75 ± 2.03 (PEF before VI, 300 V/cm). Based on statistical analysis, it was noted that the use of vacuum impregnation alone or with PEF treatment after VI did not significantly change the value of the a* parameter compared to fresh raw material. However, PEF treatment at 212.5 or 300 V/cm applied before the main process resulted in statistically significant changes, indicating a higher contribution of red color in these two variants than in the other samples.

The value of the parameter b*, which indicates a change in color from blue to yellow, ranged from 18.41 ± 3.20 to 25.87 ± 0.87. Regardless of the treatment used, the values of this parameter were not statistically significantly different from either the fresh sample or the sample subjected to vacuum impregnation alone.

Concerning the total color difference (∆E), the higher the value of ∆E, the greater the change in color is between the compared materials [59,60]. The ∆E values ranged from 25.80 to 34.56 and the values were significantly higher than 5, which means a noticeable color difference. When PEF treatment was applied, the color changes were not statistically different than in the sample treated only with vacuum impregnation.

**Figure 5:**
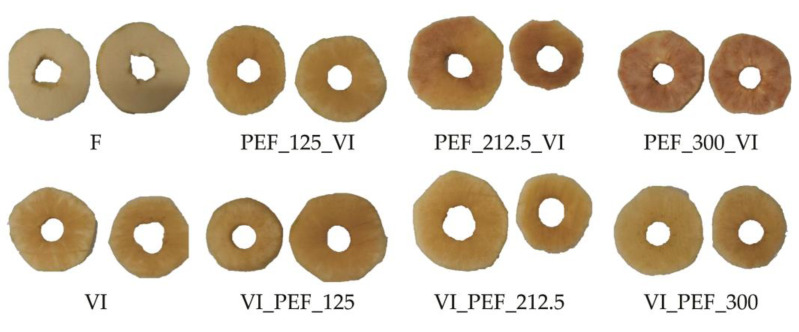
Macroscopic photographs of fresh and vacuum-impregnated apples obtained with or without the use of PEF treatment at different intensities (125, 212.5, 300 V/cm) applied before or after vacuum impregnation.

The browning index BI is a measure reflecting the browning process of the apple flesh. In fact, in most of the tested samples, the value of the a* increased, while the value of b* decreased, which may suggest the occurrence of a browning reaction [44]. This process is the result of the activity of the enzymes present in the apple tissue, which can cause negative changes in appearance and sensory characteristics. This index is calculated based on the parameters L*, a* and b* and characterizes the intensity of the brown color [61]. For fresh fruit, the browning index had a value of 34.60 ± 2.92, and after processing the apples, the index value significantly increased by 30.1 to 121.9%. The value of the browning index when VI was used was 53.13 ± 16.70 (an increase of 53.5% in relation to fresh fruit). The greatest increase in brown color intensity was observed when PEF treatments of 212.5 or 300 V/cm were applied before vacuum impregnation (81.2 and 121.9% increase, respectively). The increase in brown color intensity of these variants can be seen in the macroscopic images of the samples (Figure 5). This was most likely due to the significant damage to the structure caused by irreversible electroporation during the PEF treatment. Depending on the applied parameters, PEF treatment can affect both the increase and decrease of enzyme activity [62]. Because of that, it is recommended to use a lower electric field strange, which assures reversible electroporation.

#### 3.2.3. Changes in Apples Texture and Structure

**Figure 6:**
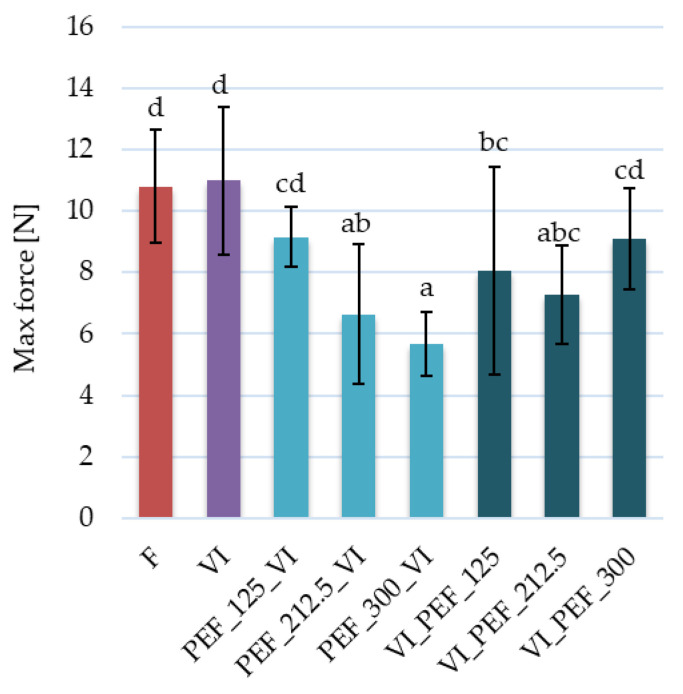
Hardness of fresh and vacuum-impregnated apples obtained with or without the use of PEF treatment of different intensities (125, 212.5, 300 V/cm) applied before or after vacuum impregnation. The same letters above the columns indicate homogeneous groups that do not differ statistically from each other (α = 0.05).

The effect of the applied treatments on the texture of the apple tissue was determined based on hardness, defined as the maximum force required to obtain a given deformation. For the fresh apple, the average hardness was 10.80 N and as a result of vacuum impregnation, this value remained almost unchanged (Figure 6). No statistically significant differences in apple hardness as a result of vacuum impregnation were also observed by Tappi et al. [6] and Derossi et al. [43]. However, from Figure 6, it can be seen that no statistically significant differences may be due to the fact that hardness was characterized by a relatively large variation for each sample (values of standard deviation ranged from 10.7 to 42% of the mean value). These high values of standard deviation are related to the method of measurement as well as on the homogeneity of the tested samples. Similar large variation in hardness was noted for apples vacuum impregnated in sucrose solution with ascorbic acid, calcium lactate or a combination of both [44].

PEF treatment of the apple tissue resulted in a significant decrease in the hardness of most samples. Similar results were obtained by Mashkour et al. [46], in which the effect of PEF treatment on the hardness of vacuum-impregnated potatoes was analyzed. Their results showed that PEF treatment significantly affected the reduction in hardness. Moreover, the researchers observed that PEF treatment of higher intensity led to a greater reduction in hardness, which is in agreement with the results obtained in our research. The reason for the hardness reduction as a result of PEF treatment could be related to significant damage to the plant structure. Texture parameters depend not only on the size of the intercellular spaces but also on the cell size, shape and packing, cell wall thickness and strength, adhesion of the middle lamellae and cell turgor [63].

**Figure 7:**
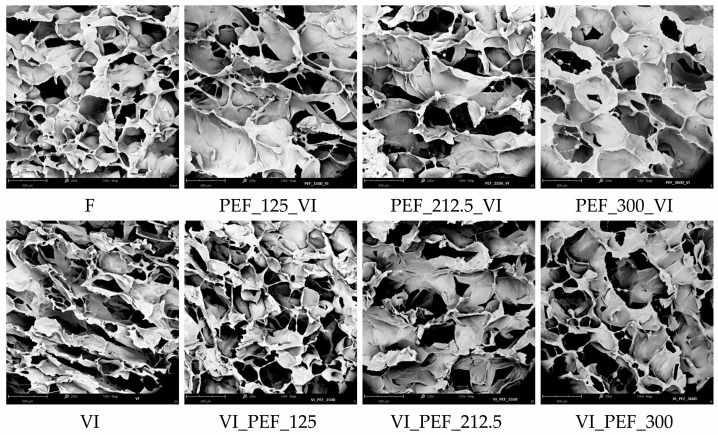
Microstructural changes of fresh and vacuum-impregnated apples obtained with or without the use of PEF treatment of different intensities (125, 212.5, 300 V/cm) applied before or after vacuum impregnation, magnification 200×.

The implemented VI and PEF processes caused significant changes in the cellular microstructure, as can be seen in images taken with a scanning electron microscope (Figure 7). The fresh apples were characterized by a porous internal structure. The material consisted of many cells tightly bound together, and the intercellular spaces were small. A similar cellular structure was observed for fresh pear [64] and strawberry [65]. Vacuum impregnation of apple tissue caused cell deformation and narrowing of the space between pores, which was also observed by Ertek et al. [66] for VI of strawberries. The change in shape and reduction of the cells can be explained by the loss of native fluid from the tissue, which occurs due to the cracking of the cell walls due to impregnation.

As a result of the PEF pre-treatment, the formation of large pores into which substances from the impregnating solution penetrated was observed. The material obtained using PEF pre-treatment with an intensity of 125 V/cm seemed to be more filled with impregnating solution than the materials obtained using PEF pre-treatment with higher intensities. In addition, in all variants in which PEF pre-treatment was used, a more regular, closer to circular pore shape and less deformation of the tissue can be observed than when only VI was performed. When PEF treatment was applied after VI, other changes in the microstructure of the apples were observed. When PEF at 125 V/cm was used the pores were relatively small in size, their shape was regular, and they differed slightly from the shape of the pores in the fresh sample. When PEF treatments at 212.5 or 300 V/cm were applied after VI of the tissue with the solution, there was significant tissue damage and the pores were very large compared to other samples. The effect of PEF treatment on the structure of porous tissue is not fully understood, and due to the different structures of different raw materials, PEF treatment can cause different changes depending on the material. For example, the tissue structure of sweet potato inside the product remained mostly intact after PEF treatment at 0.5 kV/cm and was not significantly different from that of fresh material [67]. On the other hand, the dried plums pre-treated with PEF were characterized by significant changes in microstructure compared to the dried material not treated with PEF. Similarly, as in the present study, higher-intensity PEF treatment resulted in the greater destruction of the material structure [68].

### 3.3. The Effect of Vacuum Impregnation of Apples in Aloe Vera Juice on the Change of Chemical Properties

#### 3.3.1. Bioactive Compounds and Antioxidant Activity in Apple Tissue

**Figure 8:**
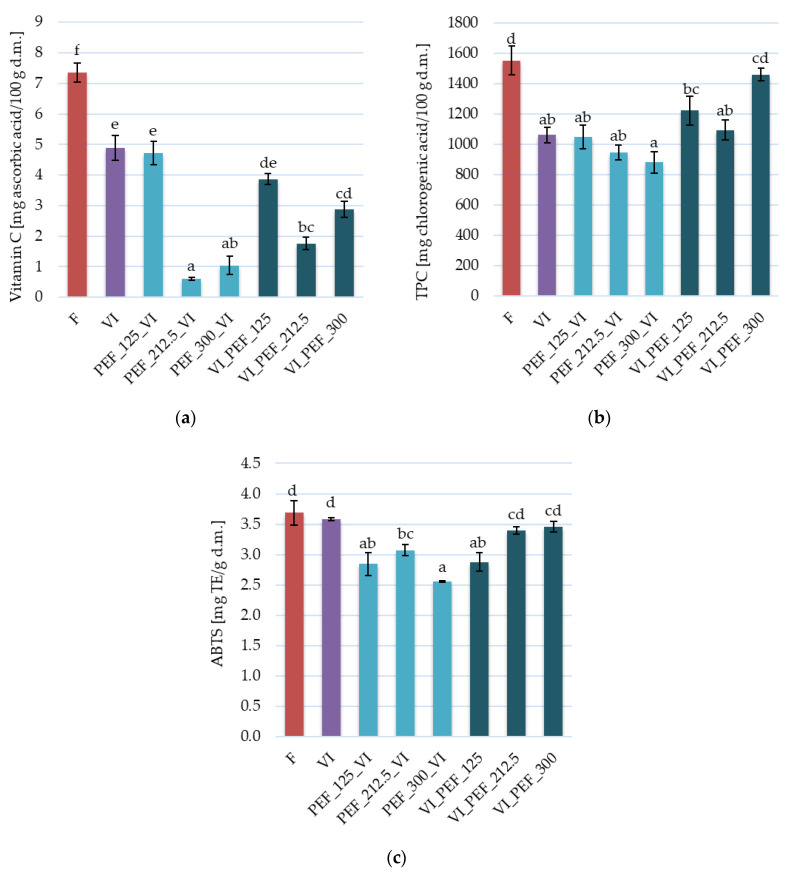
Vitamin C content (**a**), total polyphenol content (TPC) (**b**) and antioxidant activity (**c**) in fresh and vacuum-impregnated apples obtained with or without the use of PEF treatment of different intensities (125, 212.5, 300 V/cm) applied before or after vacuum impregnation. The same letters above the columns indicate homogeneous groups that do not differ statistically from each other (α = 0.05).

Figure 8 shows the vitamin C content, total polyphenols content and antioxidant activity of the apple samples. The vitamin C content of the fresh fruit was 7.36 ± 0.32 mg/100 g d.m., as a result of vacuum impregnation, this value significantly decreased to 4.89 ± 0.4 mg/100 g d.m. (Figure 8a). Furthermore, the application of high-intensity PEF treatment (212.5 or 300 V/cm) before or after VI resulted in a statistically significant reduction in the vitamin C content in the apples in comparison to the VI apples. This was probably due to leakage of vitamin C from the cells of the apple tissue caused by irreversible electroporation [69]. However, it should be noted that PEF treatment may cause also the deactivation of enzymes [70] that are responsible for the degradation of ascorbic acid [71]. On the other hand, the PEF treatment of 125 V/cm, which could induce reversible electroporation, applied before or after the main process did not result in statistically significant changes in relation to the vacuum impregnation alone. Similarly, Ciurzyńska et al. [72] studied the effect of PEF pre-treatment with different parameters prior to drying of apples. The researchers observed that the use of PEF treatments at 1, 3.5 and 6 kJ/kg before drying resulted in lower vitamin C content in the dried tissue than in the case of samples without any pre-treatment. In addition, PEF treatment of 1 kJ/kg enabled the lowest losses of this compound among PEF-treated materials.

In the case of total polyphenol content, fresh apples contained 1553 ± 93 mg of chlorogenic acid per 100 g of dry matter (Figure 8b). Vacuum impregnation alone led to a statistically significant decrease in polyphenol content by 31.6%. The decrease in the TPC of the apple tissue as a result of VI can be explained by the fact that the TPC of the apple cell juice, which leaks during the process, is higher than that of the aloe vera juice solution used in this experiment. On the contrary, when VI is carried out in a solution rich in bioactive compounds, there can be an increase in their content in the impregnated tissue. This was obtained also by Dinçer [73] when using a solution of hibiscus juice, it was rich in anthocyanins for vacuum-impregnating apples.

When PEF treatment was applied to the apple tissue, the TPC decreased significantly with regard to the fresh sample (excluding the use of PEF at 300 V/cm applied after VI) by up to 43.2%. It was noted that the application of PEF treatment prior to vacuum impregnation resulted in a greater reduction in total polyphenol content when the higher PEF intensity was used. This can be linked with the possibility of the formation of free radicals and reactive oxygen species during the PEF treatment as a response to stress [74]. The decrease in total polyphenol content in vacuum-impregnated apples after PEF pre-treatment is thought to be due to changes in structure, which may have increased cell juice leakage before or during the first phase of impregnation or decreased penetration of the impregnating solution into the voids during the second phase of impregnation. The opposite direction of change in TPC was observed when PEF treatment was carried out after the vacuum impregnation process. This indicates different changes in the microstructure of the fruit caused by the treatments used.

The antioxidant activity of apples after VI was 3.68 ± 0.20 mg Trolox/g d.m. (Figure 8c) and did not differ significantly from fresh apples, for which it was 3.58 ± 0.02 mg Trolox/g d.m. Furthermore, no statistically significant differences in antioxidant activity were observed between fresh and vacuum-impregnated apples using PEF at 212.5 V/cm—before VI, 212.5 or 300 V/cm—after VI. On the other hand, the other samples showed a decrease in antioxidant activity in the range of 19.2–43.2%. This phenomenon may be explained by the bidirectional mass transfer during the VI process. In the first step of the VI process, there is a partial loss of bioactive compounds from the porous tissue, while in the second step of the process, bioactive substances can penetrate into the tissue along with the impregnating solution. Owing to the changes in texture that occur as a result of PEF treatment, mass transfer can increase in both the first and second stages of VI, and additionally, mass transfer also occurs during PEF treatment itself. The inhomogeneous PEF effect can lead to different changes in the antioxidant activity of treated samples. Genovese et al. [30] analyzed the effects of PEF treatment with a specific energy consumption of 1.92 kJ/kg in combination with osmotic dehydration on the properties of dried kiwifruit. For all the drying temperatures used (50, 60 and 70 °C), the PEF pre-treatment gave the highest antioxidant potential of the kiwifruit samples.

#### 3.3.2. Fourier Infrared Spectroscopy (FTIR) of Apple Tissue

Fourier transform infrared spectroscopy (FTIR) was used to determine the molecular structure and chemical composition of analyzed materials. Infrared radiation is absorbed at different frequencies, which are related to the vibrational energy between atoms in a molecule [75].

**Figure 9:**
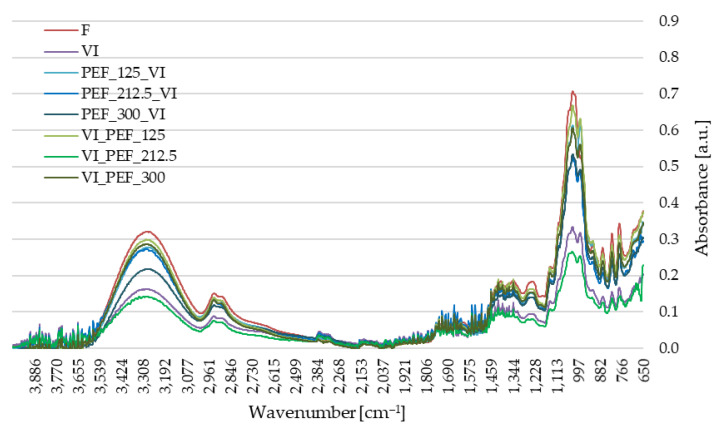
FTIR spectra of fresh and vacuum-impregnated apples obtained with or without the use of PEF treatment of different intensities (125, 212.5 and 300 V/cm) applied before or after vacuum impregnation.

Figure 9 presents the FTIR spectra of the tested samples. The spectra show the correlation between the intensity of the absorbance of the radiation and its energy expressed as a wavenumber (cm^−1^). The tested materials showed a similar peak distribution, while the peaks differed in height between the different materials. The presence of the same functional groups in the analyzed materials is due to the use of the same raw materials to obtain the individual variants, while different processing methods of the raw materials have resulted in changes in structure and chemical composition, resulting in different peak heights. The greatest differences between the materials were observed in the range 3500–2800 cm^−1^ (O-H group area) and 1800–600 cm^−1^ (C-H group area) [76].

Pulsed electric field treatment applied prior to VI generally resulted in the observed peaks being lower than for fresh material but higher than for material treated alone with vacuum impregnation. Vibrations visible in the range 4000–3600 cm^−1^ indicated the presence of water molecules. Subsequently, the characteristic of broad band in the 3600–3000 cm^−1^ region is related to the presence of hydroxyl groups, which may originate from alcohols, phenols or carboxylic acids [77]. Absorbance peaks around 2950 and 2855 cm^−1^ indicate asymmetric stretching and deformation of the -CH_2_ and -CH_3_ alkyl groups. In this region, the highest intensity spectra were recorded for fresh and VI apples with PEF treatment at 125 V/cm applied after VI. The bands in the 1750–1650 cm^−1^ region relate to vibrations of the carbonyl group (C=O) and are characteristic of aldehydes, esters and aromatic acids. Absorbance peaks in the range 1450–1300 cm^−1^ also indicated the presence of alkanes in the fruit [78]. Strong stretching vibrations for groups: (C-O) and (C-C) were recorded in the 900–1150 cm^−1^ region, e.g., for different saccharide groups such as glucose, fructose and sucrose [75]. The highest intensity in this range was observed for fresh and vaccum-impregnated material with PEF treatment at 125 V/cm intensities applied before or after VI as well as with 300 V/cm intensities applied after VI. The region below 900 cm^−1^ relates to the conformational changes of the material, where organic compounds exhibit unique molecular oscillations [79].

#### 3.3.3. Thermogravimetric (TGA) Changes in Apple Tissue

**Figure 10:**
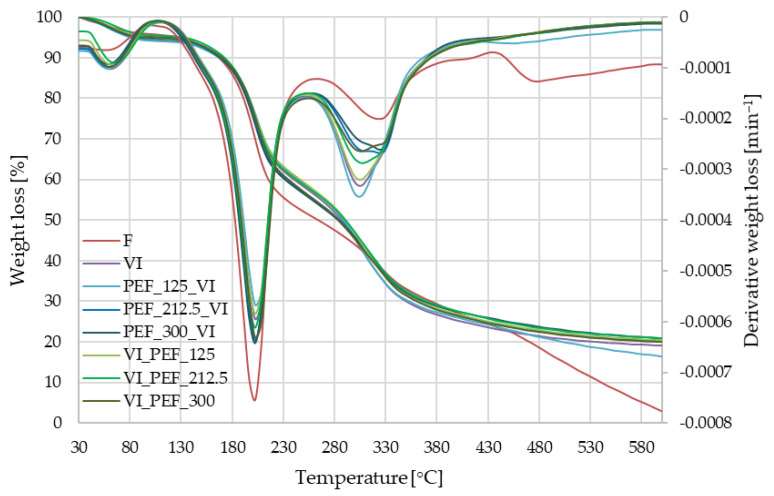
Phases of thermal degradation analysis (TGA and DTG) of fresh and vacuum-impregnated apples obtained with or without the use of PEF treatment of different intensities (125, 212.5 and 300 V/cm) applied before or after vacuum impregnation.

Figure 10 shows the temperatures and weight losses by phase of thermal degradation for fresh, VI apples without additional treatments and with PEF treatment. All VI materials showed four phases of thermal degradation.

The mass losses observed in phase I (30–110 °C) of the degradation were 4.5–6.1%. These losses were mainly due to the loss of water and volatiles. As the materials were previously lyophilized, the weight losses in this phase were relatively low, with the vacuum-impregnate apples being higher (4.7–6.1%) than untreated apples (4.5%).

In phase II (110–250 °C), mass losses in the range of 33.3–43.5% were recorded and these were the highest losses obtained by thermogravimetric analysis. Thermal degradation of fructose (119–165 °C), glucose (150–200 °C) and sucrose (170–212 °C) occurs in this phase [80]. The weight loss from fresh tissue reached 43.5%, while the weight loss from tissue only vacuum-impregnated was 36%. Weight loss from tissue subjected to pulsed electric field and vacuum impregnation ranged from 34.8 to 38.5%. From preliminary studies, it is known that the fresh apple contained 4 g sucrose, 12.3 g glucose and 41.1 g fructose in 100 g of dry matter. Based on the results of the thermogravimetric analysis, it can be assumed that there was a loss of mono- and disaccharides from the tissue as a result of the vacuum impregnation.

In phase III (250–440 °C), weight losses were also high, varying from 28.4 to 36.8%. The smallest loss in this phase (28.4%) was recorded for fresh apples. For vacuum-impregnated tissue, the weight loss in phase III was considerably higher (35.1%). The effect of PEF treatment was inconclusive, with less weight loss occurring in most variants with its use than with the use of VI alone. In phase IV (440–600 °C), weight losses for VI samples ranged from 4.0 to 7.2% and 20.6% for the non-impregnated sample.

In phase III (250–440 °C), polysaccharide degradation begins and continues in phase IV. The polysaccharides present in apples—hemicellulose, cellulose and lignin are degraded at temperatures of 220–315 °C, 250–450 °C and 320–450 °C, respectively [77,81]. The chemical composition of aloe vera juice should also be noted. It contains more than 75 biologically active components such as vitamins, minerals, monosaccharides, polysaccharides (glucomannan and acemannan), amino acids, anthraquinones, saponins, phytosterols, phenolic compounds and salicylic acids. The main phytonutrients are polysaccharides and anthraquinones [82]. The most important polysaccharide of aloe vera is acemannan, the degradation of which mainly occurs at temperatures between 200 and 600 °C [83]. Significantly higher weight losses in phase III for apples impregnated with the aloe vera juice solution compared to non-impregnated apples may indicate that the solution has effectively penetrated the pores of the apple tissue as a result of impregnation.

### 3.4. Cluster Analysis and Principal Components Analysis Results

**Figure 11:**
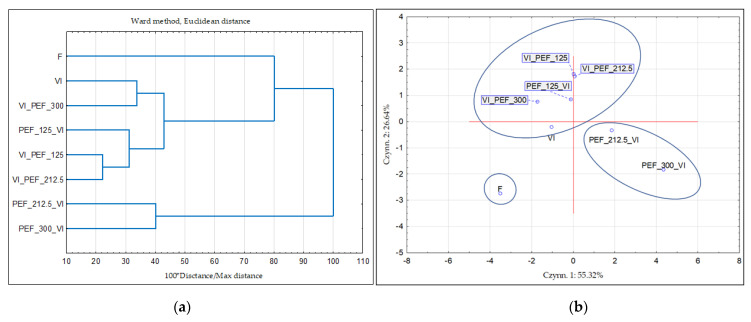
Cluster analysis (**a**) and Principal Components Analysis (PCA) (**b**) of fresh and vacuum-impregnated apples obtained with or without the use of PEF treatment of different intensities (125, 212.5 and 300 V/cm) applied before or after vacuum impregnation.

The results of the cluster analysis (CA) and Principal Components Analysis (PCA) are presented in Figure 11. The CA and PCA analyses were conducted taking into account water content, water activity, color parameters L*, a*, b*, BI; hardness, total polyphenol content, vitamin C content and antioxidant activity. The results of the analysis made it possible to distinguish three large groups (clusters). Cluster 1 included only a fresh sample, while the second group stands for the sample subjected to the VI applied alone and with the application of PEF used as post-treatment and only in one case for pre-treatment when the lowest electric field strength (125 V/cm) was used. This shows that both VI and PEF treatment caused changes in plant tissue. Another cluster contains two samples obtained by PEF treatment with an intensity of 212.5 or 300 V/cm applied before vacuum impregnation. This means that higher intensities of PEF treatment caused more pronounced changes in the structure and properties of the apple tissue, which cannot be restored during the vacuum impregnation process.

## 4. Conclusions

The vacuum impregnation process resulted in obtaining a product with a higher water content and a significant difference in color compared to the fresh material. The weight of the sample impregnated with aloe vera juice increased by over 24%. Furthermore, there were observed significant changes in the structure of the material and partial loss of cell viability, which can be related to a decrease in the polyphenols and vitamin C content. However, the antioxidant activity for the VI sample did not change statistically.

Due to the mechanism of the PEF treatment, it was observed several negative effects such as changes in the microstructure of the plant tissue as well as partial (125 V/cm) or complete (212.5, 300 V/cm) loss of cell viability, which can be linked to the texture altering (softening).

As a result of PEF treatment, applied both before and after vacuum impregnation, the hardness of the tissue decreases significantly, which is associated with damage to the structure, leakage from cells and low efficiency of tissue saturation with the impregnating solution. This is probably related to the greater leaking from the apple tissue than the force caused by the pressure forcing the surrounding aloe vera juice into the tissue due to the vacuum impregnation process. The reduced content of bioactive substances after PEF treatments results from changes in the structure of the material, which on the one hand may support mass exchange, and on the other—cause the loss of nutritionally valuable substances. The reduction of bioactive compounds, including vitamin C content (PEF 212.5 or 300 V/cm applied before and after vacuum impregnation) and antioxidant activity (PEF 125 or 300 V/cm applied before vacuum impregnation or 125 V/cm after vacuum impregnation) was observed. Whereas total polyphenol content was statistically unchanged or higher in comparison to untreated vacuum-impregnated samples. The use of pulsed electric field treatment reduces the effectiveness of the vacuum impregnation process, so the PEF treatment is not recommended to be used before or after the vacuum impregnation process.

## Figures and Tables

**Table 1:** Electrical capacitance and resistance of the samples subjected to the PEF treatment and the average was calculated from at least 8 repetitions.

Treatments *	Electrical Capacitance [nF]		Resistance [kΩ]	
	Average	SD	Average	SD
F	3.89	0.47	5.14	0.47
PEF_125	4.14	0.63	4.77	0.63
PEF_212.5	4.96	0.50	4.90	0.36
PEF_300	5.27	0.32	2.97	0.50

* F—fresh sample, PEF_x—means the pulsed electric field treatment with the use of electric field strength equal to 125, 212.5 or 300 V/cm.

**Table 2:** Code and types of the obtained samples.

Code	VI		PEF Intensities [V/cm]
	Pre-Processing	Post-Processing	
F	−		
VI	+		
PEF_125_VI		+	125
PEF_212.5_VI		+	212.5
PEF_300_VI		+	300
VI_PEF_125	+		125
VI_PEF_212.5	+		212.5
VI_PEF_300	+		300

**Table 3:** Color parameters of fresh and vacuum-impregnated apples obtained with or without the use of PEF treatment at different intensities (125, 212.5 and 300 V/cm) applied before or after vacuum impregnation.

Sample	L*	a*	b*	ΔE	BI
F	78.63 ± 2.22 ^c^*	0.92 ± 0.23 ^a^	23.16 ± 1.10 ^abc^		33.56 ± 2.16 ^a^
VI	50.35 ± 6.55 ^ab^	0.77 ± 1.10 ^a^	20.99 ± 6.09 ^abc^	28.93 ± 6.61 ^abc^	46.02 ± 7.14 ^bc^
PEF_125_VI	45.36 ± 2.73 ^a^	1.34 ± 1.18 ^a^	18.49 ± 3.02 ^a^	33.64 ± 2.85 ^bc^	55.32 ± 9.69 ^cd^
PEF_212.5_VI	53.09 ± 2.18 ^b^	4.31 ± 2.24 ^b^	23.54 ± 1.69 ^bc^	25.80 ± 2.33 ^a^	59.25 ± 7.25 ^d^
PEF_300_VI	52.25 ± 2.53 ^b^	7.75 ± 2.03 ^c^	25.87 ± 0.87 ^c^	27.44 ± 2.90 ^ab^	79.60 ± 6.97 ^e^
VI_PEF_125	44.43 ± 5.43 ^a^	1.06 ± 0.51 ^a^	19.13 ± 4.29 ^ab^	34.56 ± 5.65 ^c^	52.05 ± 6.05 ^cd^
VI_PEF_212.5	47.62 ± 5.41 ^ab^	1.00 ± 1.72 ^a^	20.43 ± 5.00 ^ab^	31.40 ± 5.70 ^abc^	48.49 ± 9.19 ^bc^
VI_PEF_300	49.30 ± 4.37 ^ab^	0.10 ± 1.01 ^a^	18.41 ± 3.20 ^a^	29.76 ± 4.73 ^abc^	41.54 ± 5.35 ^ab^

* The same letters (a–e) in columns indicate homogeneous groups that do not differ statistically from each other (α = 0.05).

## Data Availability

The data presented in this study are available on request from the corresponding author.

## References

[1] Saleena P., Jayashree E., Anees K. (2023). A Comprehensive Review on Vacuum Impregnation: Mechanism, Applications and Prospects. Food Bioprocess Technol..

[2] Fito P., Andrés A., Chiralt A., Pardo P. (1996). Coupling of Hydrodynamic Mechanism and Deformation-Relaxation Phenomena during Vacuum Treatments in Solid Porous Food-Liquid Systems. J. Food Eng..

[3] Fito P., Chiralt A., Betoret N., Gras M., Cháfer M., Martínez-Monzó J., Andrés A., Vidal D. (2001). Vacuum impregnation and osmotic dehydration in matrix engineering: Application in functional fresh food development. J. Food Eng..

[4] Mújica-Paz H., Valdez-Fragoso A., López-Malo A., Palou E., Welti-Chanes J. (2003). Impregnation and osmotic dehydration of some fruits: Effect of the vacuum pressure and syrup concentration. J. Food Eng..

[5] Gras M., Vidal-Brotóns N., Betoret A., Chiralt, Fito P. (2002). The response of some vegetables to vacuum impregnation. Innov. Food Sci. Emerg. Technol..

[6] Tappi S., Tylewicz U., Romani S., Dalla Rosa M., Rizzi F., Rocculi P. (2017). Study on the quality and stability of minimally processed apples impregnated with green tea polyphenols during storage. Innov. Food Sci. Emerg. Technol..

[7] Tappi S., Tylewicz U., Romani S., Siroli L., Patrignani F., Dalla Rosa M., Rocculi P. (2016). Optimization of Vacuum Impregnation with Calcium Lactate of Minimally Processed Melon and Shelf-Life Study in Real Storage Conditions. J. Food Sci..

[8] Erihemu, Hironaka K., Oda Y., Koaze H. (2014). Iron enrichment of whole potato tuber by vacuum impregnation. LWT.

[9] Hironaka K., Kikuchi M., Koaze H., Sato T., Kojima M., Yamamoto K., Yasuda K., Mori M., Tsuda S. (2011). Ascorbic acid enrichment of whole potato tuber by vacuum-impregnation. Food Chem..

[10] Park S.I., Kodihalli I., Zhao Y. (2005). Nutritional, sensory, and physicochemical properties of vitamin E- and mineral-fortified fresh-cut apples by use of vacuum impregnation. J. Food Sci..

[11] de Oliveira P.M., Ramos A.M., Martins E.M.F., Vieira É.N.R., de Souza Soares A., de Noronha M.C. (2017). Comparison of vacuum impregnation and soaking techniques for addition of the probiotic Lactobacillus acidophilus to minimally processed melon. Int. J. Food Sci. Technol..

[12] Castagnini J.M., Tappi S., Tylewicz U., Laghi L., Rocculi P. (2022). Study of Water Distribution, Textural and Colour Properties of Cold Formulated and Air-Dried Apple Snacks. Foods.

[13] González-Pérez J.E., Jiménez-González O., Ramírez-Corona N., Guerrero-Beltrán J.A., López-Malo A. (2022). Vacuum impregnation on apples with grape juice concentrate: Effects of pressure, processing time, and juice concentration. Innov. Food Sci. Emerg. Technol..

[14] Betoret N., Puente L., Díaz M.J., Pagán M.J., García M.J., Gras M.L., Martínez-Monzó J., Fito P. (2003). Development of probiotic-enriched dried fruits by vacuum impregnation. J. Food Eng..

[15] Betoret E., Betoret N., Arilla A., Bennár M., Barrera C., Codoñer P., Fito P. (2012). No invasive methodology to produce a probiotic low humid apple snack with potential effect against Helicobacter pylori. J. Food Eng..

[16] Derossi A., Ricci I., Fiore Ricci A.G., Severini C. (2018). Apple slices enriched with aloe vera by vacuum impregnation. Ital. J. Food Sci..

[17] Mitra A., Singh M., Banga A., Pandey J., Tripathi S.S., Singh D. (2023). Bioactive compounds and therapeutic properties of Aloe vera—A review. Plant Sci. Today.

[18] Deep A., Kumar D., Bansal N., Narasimhan B., Marwaha R.K., Sharma P.C. (2023). Understanding mechanistic aspects and therapeutic potential of natural substances as anticancer agents. Phytomed. Plus.

[19] Taukoorah U., Mahomoodally M.F. (2016). Crude Aloe vera Gel Shows Antioxidant Propensities and Inhibits Pancreatic Lipase and Glucose Movement in Vitro. Adv. Pharmacol. Sci..

[20] Kumar S.V., Kumar S.P., Rupesh D., Nitin K. (2010). Aloe vera: A Potential Herb and its Medicinal Importance. J. Chem. Pharm. Res..

[21] He Q., Changhong L., Kojo E., Tian Z. (2005). Quality and safety assurance in the processing of aloe vera gel juice. Food Control.

[22] Kumar S., Kalita S., Das A., Kumar P., Singh S., Katiyar V., Mukherjee A. (2022). Aloe vera: A contemporary overview on scope and prospects in food preservation and packaging. Prog. Org. Coat..

[23] Donsì F., Ferrari G., Pataro G. (2010). Applications of pulsed electric field treatments for the enhancement of mass transfer from vegetable tissue. Food Eng. Rev..

[24] Puértolas E., Luengo E., Álvarez I., Raso J. (2012). Improving mass transfer to soften tissues by pulsed electric fields: Fundamentals and applications. Annu. Rev. Food Sci. Technol..

[25] Li J., Dadmohammadi Y., Li P., Madarshahian S., Abbaspourrad A. (2022). Generation of garlic flavor after frying by infusing alliin into potato strips using pulsed electric field and assisted infusion methods. Food Chem..

[26] Velickova E., Tylewicz U., Dalla Rosa M., Winkelhausen E., Kuzmanova S., Romani S. (2018). Effect of pulsed electric field coupled with vacuum infusion on quality parameters of frozen/thawed strawberries. J. Food Eng..

[27] Shiekh K.A., Benjakul S., Gulzar S. (2021). Impact of pulsed electric field and vacuum impregnation with Chamuang leaf extract on quality changes in Pacific white shrimp packaged under modified atmosphere. LWT.

[28] Tylewicz U., Lundin P., Cocola L., Dymek K., Rocculi P., Svanberg S., Dejmek P., Gόmez Galindo F. (2012). Gas in scattering media absorption spectroscopy (GASMAS) detected persistent vacuum in apple tissue after vacuum impregnation. Food Biophys..

[29] Association of Official Analytical Collaboration International (2002). Official Methods of Analysis of AOAC International.

[30] Genovese J., Tylewicz U., Mannozzi C., Castagnini J.M., Romani S., Rocculi P., Dalla Rosa M. (2022). Kiwifruit waste valorisation through innovative snack development. Acta Hortic..

[31] Cerezal-Mezquita P., Bugueño-Muñoz W. (2022). Drying of Carrot Strips in Indirect Solar Dehydrator with Photovoltaic Cell and Thermal Energy Storage. Sustainability.

[32] Paula A.M., Conti-Silva A.C. (2014). Texture profile and correlation between sensory and instrumental analyses on extruded snacks. J. Food Eng..

[33] Biswas M.S., Terada R., Mano J. (2020). Inactivation of Carbonyl-Detoxifying Enzymes by H_2_O_2_ Is a Trigger to Increase Carbonyl Load for Initiating Programmed Cell Death in Plants. Antioxidants.

[34] Bochnak-Niedźwiecka J., Szymanowska U., Świeca M. (2022). The Protein-Rich Powdered Beverages Stabilized with Flax Seeds Gum—Antioxidant and Antiproliferative Properties of the Potentially Bioaccessible Fraction. Appl. Sci..

[35] Beya M.M., Netzel M.E., Sultanbawa Y., Smyth H., Hoffman L.C. (2023). Kakadu plum (Terminalia ferdinandiana) bioactivity against spoilage microorganisms and oxidative reactions in refrigerated raw beef patties under modified atmosphere packaging. Meat Sci..

[36] Matys A., Dadan M., Witrowa-Rajchert D., Parniakov O., Wiktor A. (2022). Response Surface Methodology as a Tool for Optimization of Pulsed Electric Field Pretreatment and Microwave-Convective Drying of Apple. Appl. Sci..

[37] Rivero-Ramos P., Unthank M.G., Sanz T., Rodrigo M.D., Benlloch-Tinoco M. (2023). Synergistic depolymerisation of alginate and chitosan by high hydrostatic pressure (HHP) and pulsed electric fields (PEF) treatment in the presence of H2O2. Carbohydr. Polym..

[38] Mikus M., Galus S., Ciurzyńska A., Janowicz M. (2021). Development and characterization of novel composite films based on soy protein isolate and oilseed flours. Molecules.

[39] Panayampadan A.S., Alam M.S., Aslam R., Kaur J. (2022). Vacuum Impregnation Process and Its Potential in Modifying Sensory, Physicochemical and Nutritive Characteristics of Food Products. Food Eng. Rev..

[40] Martínez-Monzó J., Barat J.M., González-Martínez C., Chiralt A., Fito P. (2000). Changes in thermal properties of apple due to vacuum impregnation. J. Food Eng..

[41] Yılmaz F.M., Ersus Bilek S. (2018). Ultrasound-assisted vacuum impregnation on the fortification of fresh-cut apple with calcium and black carrot phenolics. Ultrason. Sonochem..

[42] Paes S.S., Stringari G.B., Laurindo J.B. (2007). Effect of vacuum and relaxation periods and solution concentration on the osmotic dehydration of apples. Int. J. Food Sci. Technol..

[43] Derossi A., Francavilla M., Monteleone M., Caporizzi R., Severini C. (2021). From biorefinery of microalgal biomass to vacuum impregnation of fruit. A multidisciplinary strategy to develop innovative food with increased nutritional properties. Innov. Food Sci. Emerg. Technol..

[44] Tappi S., Velickova E., Mannozzi C., Tylewicz U., Laghi L., Rocculi P. (2022). Multi-Analytical Approach to Study Fresh-Cut Apples Vacuum Impregnated with Different Solutions. Foods.

[45] Assis F.R., Rodrigues L.G.G., Tribuzi G., de Souza P.G., Carciofi B.A.M., Laurindo J.B. (2019). Fortified apple (*Malus* spp., var. Fuji) snacks by vacuum impregnation of calcium lactate and convective drying. LWT—Food Sci. Technol..

[46] Mashkour M., Maghsoudlou Y., Kashaninejad M., Aalami M. (2018). Iron Fortification of Whole Potato Using Vacuum Impregnation Technique with a Pulsed Electric Field Pretreatment. Potato Res..

[47] Lahiri R., Adams L.B., Scollard D.M., Gillis T.P. (2016). Cultivation and Viability Determination of *Mycobacterium leprae*. International Textbook of Leprosy.

[48] Panarese V., Dejmek P., Rocculi P., Galindo F.G. (2013). Microscopic studies providing insight into the mechanisms of mass transfer in vacuum impregnation. Innov. Food Sci. Emerg. Technol..

[49] Tylewicz U., Romani S., Widell S., Gόmez Galindo F. (2013). Induction of Vesicle Formation by Exposing Apple Tissue to Vacuum Impregnation. Food Bioprocess Technol..

[50] Velickova E., Tylewicz U., Dalla Rosa M., Winkelhausen E., Kuzmanova S., Gómez Galindo F. (2013). Effect of vacuum infused cryoprotectants on the freezing tolerance of strawberry tissues. LWT.

[51] Mauro M.A., Dellarosa N., Tylewicz U., Tappi S., Laghi L., Rocculi P., Rosa M.D. (2016). Calcium and ascorbic acid affect cellular structure and water mobility in apple tissue during osmotic dehydration in sucrose solutions. Food Chem..

[52] Tylewicz U., Tappi S., Genovese J., Mozzon M., Rocculi P. (2019). Metabolic response of organic strawberries and kiwifruit subjected to PEF assisted-osmotic dehydration. Innov. Food Sci. Emerg. Technol..

[53] Tylewicz U., Tappi S., Mannozzi C., Romani S., Dellarosa N., Laghi L., Ragni L., Rocculi P., Dalla Rosa M. (2017). Effect of pulsed electric field (PEF) pre-treatment coupled with osmotic dehydration on physico-chemical characteristics of organic strawberries. J. Food Eng..

[54] de Lima M.M., Tribuzi G., de Souza J.A.R., de Souza I.G., Laurindo J.B., Carciofi B.A.M. (2016). Vacuum impregnation and drying of calcium-fortified pineapple snacks. LWT.

[55] Nawirska-Olszańska A., Pasławska M., Stępień B., Oziembłowski M., Sala K., Smorowska A. (2020). Effect of vacuum impregnation with apple-pear juice on content of bioactive compounds and antioxidant activity of dried chokeberry fruit. Foods.

[56] Hinestroza-Córdoba L.I., Barrera C., Seguí L., Betoret N. (2021). Potential use of vacuum impregnation and high-pressure homogenization to obtain functional products from lulo fruit (*Solanum quitoense* lam.). Foods.

[57] Oblitas Cruz J.F., Rojas Gutierrez E.L. (2018). Optimizing the incorporation of aloe vera in yacon slices (Smallanthus sonchifolius Poepp. & Endl.) through vacuum impregnation using response surface methodology. Ing. Univ..

[58] Nowacka M., Mannozzi C., Dalla Rosa M., Tylewicz U. (2023). Sustainable Approach for Development Dried Snack Based on Actinidia deliciosa Kiwifruit. Appl. Sci..

[59] Mokrzycki W.S., Tatol M. (2011). Colour difference ∆E—A survey Mokrzycki. Mach. Graph. Vis..

[60] Abalos R.A., Naef E.F., Aviles M.V., Gómez M.B. (2020). Vacuum impregnation: A methodology for the preparation of a ready-to-eat sweet potato enriched in polyphenols. LWT—Food Sci. Technol..

[61] Jalaee F., Fazeli A., Fatemian H., Tavakolipour H. (2011). Mass transfer coefficient and the characteristics of coated apples in osmotic dehydrating. Food Bioprod. Process..

[62] Wiktor A., Witrowa-Rajchert D. (2012). Zastosowanie pulsacyjnego pola elektrycznego do wspomagania procesów usuwania wody z tkanek roślinnych. Żywność Nauk. Technol. Jakość.

[63] Toivonen P.M.A., Brummell D.A. (2008). Biochemical bases of appearance and texture changes in fresh-cut fruit and vegetables. Postharvest Biol. Technol..

[64] Moreno J., Simpson R., Sayas M., Segura I., Aldana O., Almonacid S. (2011). Influence of ohmic heating and vacuum impregnation on the osmotic dehydration kinetics and microstructure of pears (cv. Packham’s Triumph). J. Food Eng..

[65] Moreno J., Simpson R., Baeza A., Morales J., Muñoz C., Sastry S., Almonacid S. (2012). Effect of ohmic heating and vacuum impregnation on the osmodehydration kinetics and microstructure of strawberries (cv. Camarosa). LWT.

[66] Ertek G., Taştan Ö., Baysal T. (2023). Combined use of vacuum impregnation and encapsulation technologies for phenolic enrichment of strawberries. Food Chem..

[67] Liu T., Dodds E., Leong S.Y., Eyres G.T., Burritt D.J., Oey I. (2017). Effect of pulsed electric fields on the structure and frying quality of “kumara” sweet potato tubers. Innov. Food Sci. Emerg. Technol..

[68] Rahaman A., Siddeeg A., Manzoor M.F., Zeng X.A., Ali S., Baloch Z., Li J., Wen Q.H. (2019). Impact of pulsed electric field treatment on drying kinetics, mass transfer, colour parameters and microstructure of plum. J. Food Sci. Technol..

[69] Rybak K., Wiktor A., Witrowa-Rajchert D., Parniakov O., Nowacka M. (2020). The effect of traditional and non-thermal treatments on the bioactive compounds and sugars content of red bell pepper. Molecules.

[70] Gustavo V., Barbosa-Cánovas M., Góngora-Nieto M., Pothakamury U.R., Swanson B.G., Gustavo V., Barbosa-Cánovas M., Góngora-Nieto M., Pothakamury U.R., Swanson B.G. (1999). PEF inactivation of vegetative cells, spores, and enzymes in foods. Preservation of Foods with Pulsed Electric Fields.

[71] Castro S.M., Saraiva J.A., Lopes-da-Silva J.A., Delgadillo I., Van Loey A., Smout C., Hendrickx M. (2008). Effect of thermal blanching and of high pressure treatments on sweet green and red bell pepper fruits (*Capsicum annuum* L.). Food Chem..

[72] Ciurzynska A., Trusinska M., Rybak K., Wiktor A., Nowacka M. (2023). The Influence of Pulsed Electric Field and Air Temperature on the Course of Hot-Air Drying and the Bioactive Compounds of Apple Tissue. Molecules.

[73] Dinçer C. (2022). Modeling of hibiscus anthocyanins transport to apple tissue during ultrasound-assisted vacuum impregnation. J. Food Process. Preserv..

[74] Galindo F.G., Miklavčič D. (2017). Responses of plant cells and tissues to pulsed electric field treatments. Handbook of Electroporation.

[75] Bureau S., Cozzolino D., Clark C.J. (2019). Contributions of Fourier-transform mid infrared (FT-MIR) spectroscopy to the study of fruit and vegetables: A review. Postharvest Biol. Technol..

[76] Jankovic B., Marinovic-Cincovic M., Jankovic M. (2016). TG-DTA-FTIR analysis and isoconversional reaction profiles for thermal and thermo-oxidative degradation processes in black chokeberry (Aroniamelanocarpa). Chem. Pap..

[77] Lam S.S., Liew R.K., Lim X.Y., Ani F.N., Jusoh A. (2016). Fruit waste as feedstock for recovery by pyrolysis technique. Int. Biodeterior. Biodegrad..

[78] Kowalska H., Trusinska M., Rybak K., Wiktor A., Witrowa-Rajchert D., Nowacka M. (2023). Shaping the Properties of Osmo-Dehydrated Strawberries in Fruit Juice Concentrates. Appl. Sci..

[79] Przybył K., Koszela K., Adamski F., Samborska K. (2021). Analysis to Detect Polysaccharide in Raspberry Powders. Sensors.

[80] Zlatanović S., Ostojić S., Micić D., Rankov S., Dodevska M., Vukosavljević P., Gorjanović S. (2019). Thermal behaviour and degradation kinetics of apple pomace flours. Thermochim. Acta.

[81] Cheng X.C., Cui X.Y., Qin Z., Liu H.M., Wang X.D., Liu Y.L. (2021). Effect of drying pretreatment methods on structural features and antioxidant activities of Brauns native lignin extracted from Chinese quince fruit. Process Biochem..

[82] Martínez-Burgos W.J., Serra J.L., MarsigliaF R.M., Montoya P., Sarmiento-Vásquez Z., Marin O., Gallego-Cartagena E., Paternina-Arboleda C.D. (2022). Aloe vera: From ancient knowledge to the patent and innovation landscape—A review. S. Afr. J. Bot..

[83] Nema J. (2012). Physicochemical study of acemannan polysaccharide in Aloe species under the influence of soil reaction (pH) and moisture application. Afr. J. Pure Appl. Chem..

